# Rehabilitation of the Surgical Defect Secondary to Mucormycosis With a Definitive Obturator: A Case Report

**DOI:** 10.7759/cureus.62598

**Published:** 2024-06-18

**Authors:** Vaidehi Thakare, Akansha Bansod, Arushi Beri, Surekha A Dubey, Shivangi Navghare, Nisarga R Mahajan

**Affiliations:** 1 Prosthodontics, Sharad Pawar Dental College and Hospital, Datta Meghe Institute of Higher Education and Research, Wardha, IND

**Keywords:** rehabilitation, obturator, prosthodontists, maxillectomy, mucormycosis

## Abstract

Neglect initially starts as an infection, evolving into a disease. During the COVID-19 pandemic, lifestyle changes and disturbed food intake have weakened immune systems, making individuals more susceptible to secondary infections. Mucormycosis poses a significant threat, especially during the COVID-19 pandemic when immune systems may be compromised. The impact of the disease extends beyond physical health, affecting psychological and social well-being due to the challenges, such as difficulties in mastication, speech, and swallowing post-surgery. The mention of maxillectomy highlights the severe nature of some cases, necessitating surgical intervention to remove the affected tissue. Anatomical and functional losses following surgical excision of the maxilla and surrounding tissues need to be recovered as soon as possible. After surgery, prostheses can and should be used to restore speech, mastication, and deglutition - three essential physiological activities. Thus, a patient's treatment approach should include prosthetic planning before surgical intervention and rehabilitation after the surgery. If they are carefully handled at the moment of surgery, the overall continuity created by maxilla resection includes at least the oral, nasal, and maxillary sinus cavities, which may prove to be a useful future location for prosthesis retention. As a result, the prosthodontist's role becomes crucial in assisting the surgeon in taking all necessary precautions that are beneficial to the patient.

## Introduction

Mucormycosis is an opportunistic fungal infection that often fulminates and is caused by a saprobic organism belonging to the zygomycetes class. Surgical interventions such as alveolectomy and palatectomy become inevitable when malignant and infectious lesions affect the palate, which divides the oral cavity from the nasal cavity [1]. After aspergillosis and candidiasis, it is the third most prevalent invasive fungal infection. It primarily affects individuals with low immunity and is hardly observed in otherwise healthy people [2]. Individuals with uncontrolled diabetes and impaired immune systems are at a higher risk of developing an infection that results in both soft and hard tissue necrosis. Clinically, the condition is characterized by a progressive loss of tissue infarction or necrosis and a partial loss of neurological function as a result of the organism's invasion of the blood vessels. The illness may worsen to the point where it affects the skull, which would raise the death rate. A definitive diagnosis and treatment help to overcome the disease. Mucormycosis is generally seen in India after COVID-19. The treatment of choice for patients with COVID-19 is immunosuppressive therapy. And we are well aware of the fact that immunosuppressive agents increase the risk of opportunistic infection [3]. The maxillary obturator is mainly used for the rehabilitation of the maxillary defect. Treatment may be surgical or antifungal medications, depending upon the risk factors and severity. Immunocompromised people developed mucormycosis as a result of the COVID-19 outbreak. Anti-fungal medications, surgical excision of infected tissues, and therapy of underlying metabolic problems are available forms of treatment. Usually, surgery entails completely excising the affected area. The patient is at risk for nasal twang, nasal cavity leaks, and impaired masticatory function because of these anomalies. The obturator prosthesis may form an oronasal seal in such problems. Additionally, lowering the prosthesis weight contributes to improved stability and retention.

Prosthetic rehabilitation plays a pivotal role in restoring function and aesthetics, which are crucial for the overall quality of life. The obturator acting as a barrier, not only aids in functional aspects but also helps in preventing complications like oroantral communication. It is fascinating how advancements in prosthetics enable individuals to regain a sense of normalcy amidst such challenging circumstances. This holistic approach to treatment, addressing both physical and psychosocial aspects, underscores the importance of interdisciplinary care in managing complex medical conditions like mucormycosis [2]. Obturators are prosthetic devices used to close defects resulting from maxillectomy. Three phases are involved: immediate (surgical) obturator placed at the time of surgery. An interim surgical obturator is fabricated to aid in tissue healing, typically three to four weeks post-surgery. A definitive obturator was delivered after three months for long-term rehabilitation. This comprehensive approach ensures proper planning, execution, and follow-up care for patients undergoing maxillectomy. Obturators play a crucial role in postoperative care for patients with palatal defects. The current study aimed to assess the efficacy of mastication and swallowing therapy as well as the impact of maxillary obturators on speech quality. Regaining the patient's normal daily activities as soon as feasible is important to restore oral cavity function and preserve the patient's mental health during treatment. Prosthodontists should consider the cost of the treatment and also the materials used and the manufacturing process [4].

## Case presentation

**Figure 1:**
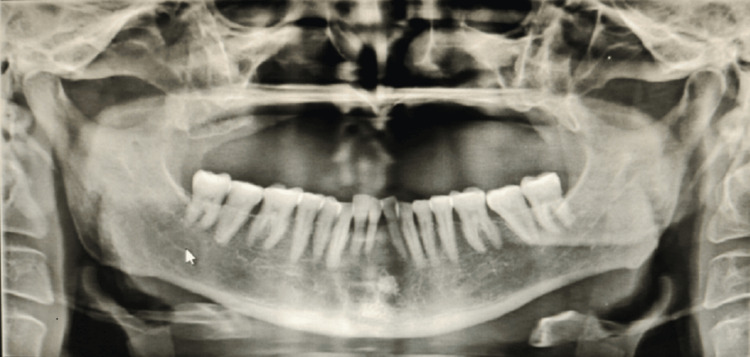
The panoramic radiograph showing the entire radiolucency in the maxillary region.

A 47-year-old patient was referred to the Department of Prosthodontics for esthetic and function concerns after undergoing surgery for mucormycosis of the maxilla. Nine months ago, a surgical excision of the anterior and posterior maxillary alveolar bones was performed. Past medical history revealed that he had a known case of COVID-19 and then of mucormycosis one year back. The patient reported to the department after post-surgery with the surgical defect, which was bilateral, extending from 14 to 16 region on the right side and 23 to 26 region from the left side and measuring above 1.5 cm x 2 cm mesiodistally and 1 cm x 1.5 cm buccolingually. The patient had undergone surgery to treat mucormycosis, that is, maxillectomy, and the surgical site was completely healed. For the previous six months, the patient had been using an interim obturator (without tooth integration) due to which the patient had difficulty in mastication and also had the loss of resonance of voice. A well-healed surgical lesion in the maxillary right and buccal vestibule that created an oroantral connection was discovered during an intraoral examination. The radiographic details showed the complete radiolucency in the maxillary region in a panoramic view (Figure 1).

**Figure 2:**
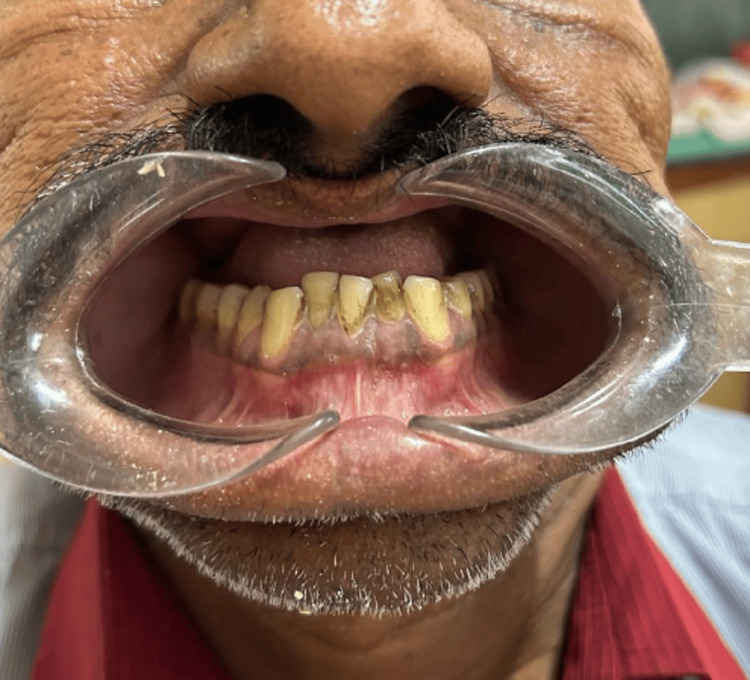
Intraoral preoperative photograph showing the mandibular teeth.

Masticatory function and esthetic function were affected. Additionally, the patient experienced discomfort while eating and swallowing, along with nasal congestion. Importantly, there was no history of purulent discharge or fever reported by the patient. On intraoral examination, the complete maxilla had been surgically removed due to mucormycosis. The treated surgical site was completely healed, and all the mandibular teeth were present (Figure 2). A well-healed surgical lesion in the maxillary right and left buccal vestibule, which created an oroantral connection, was discovered. The defect was approximately 1 cm x 1.5 cm.

**Figure 3:**
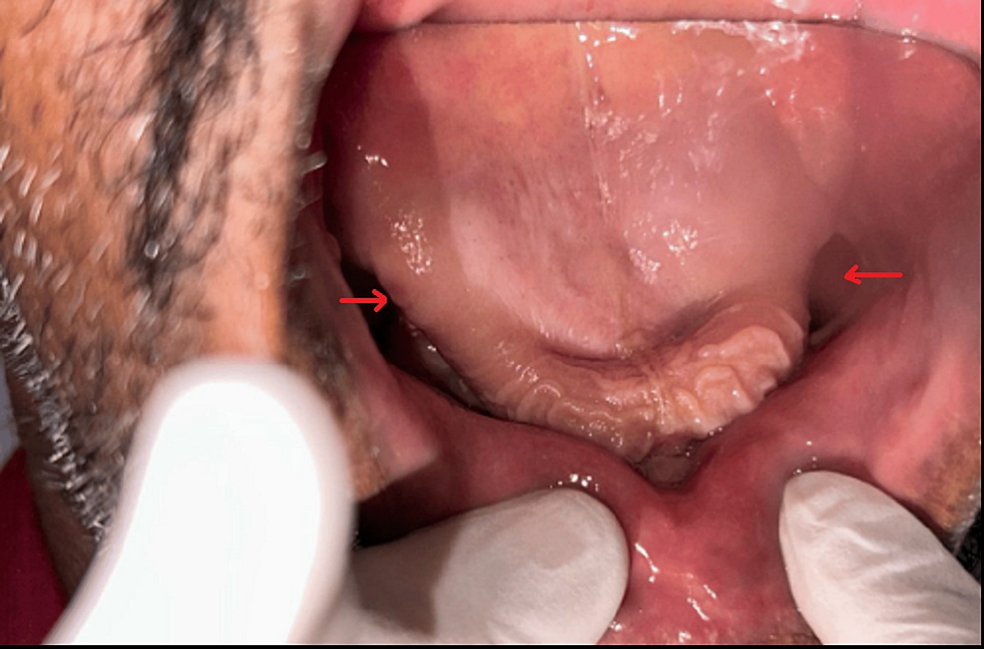
Representative figure of surgical defects in the maxilla. Arrows are indicative of oroantral communication.

The representation of the surgical defect of the maxilla suggesting an oroantral communication is shown in Figure 3. The patient was not satisfied with the interim prosthesis and wanted to get it replaced. After undergoing post-maxillectomy, the patient currently has an interim prosthesis and wishes to obtain a definitive prosthesis.

**Figure 4:**
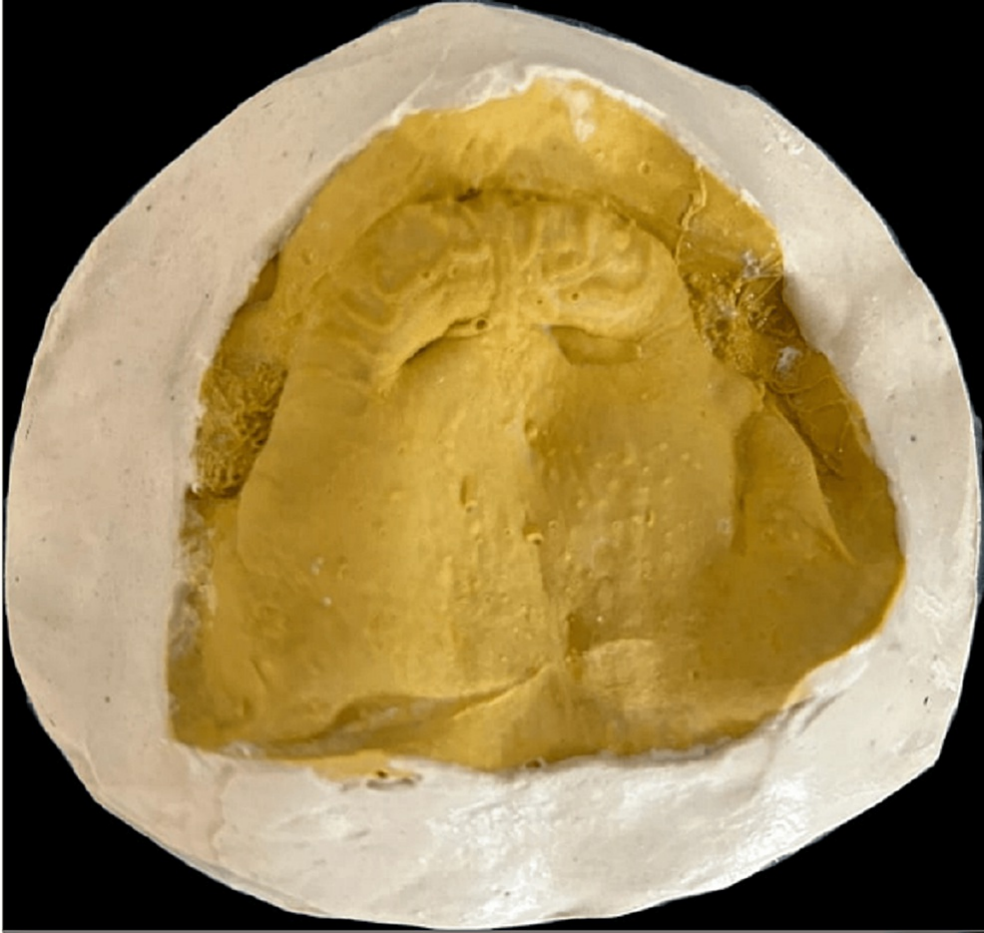
The laboratory step of the primary cast is depicted in the photograph.

Written, well-informed consent of the patient was taken before starting the procedure. The primary impression of the maxilla arch was taken by using irreversible hydrocolloid material, that is, Zelgan Advanced alginate impression material (Dentsply Sirona, Charlotte, NC). The entire maxillary defect was covered by the primary impression, which was then filled with type III dental stone to create the primary cast (Figure 4).

**Figure 5:**
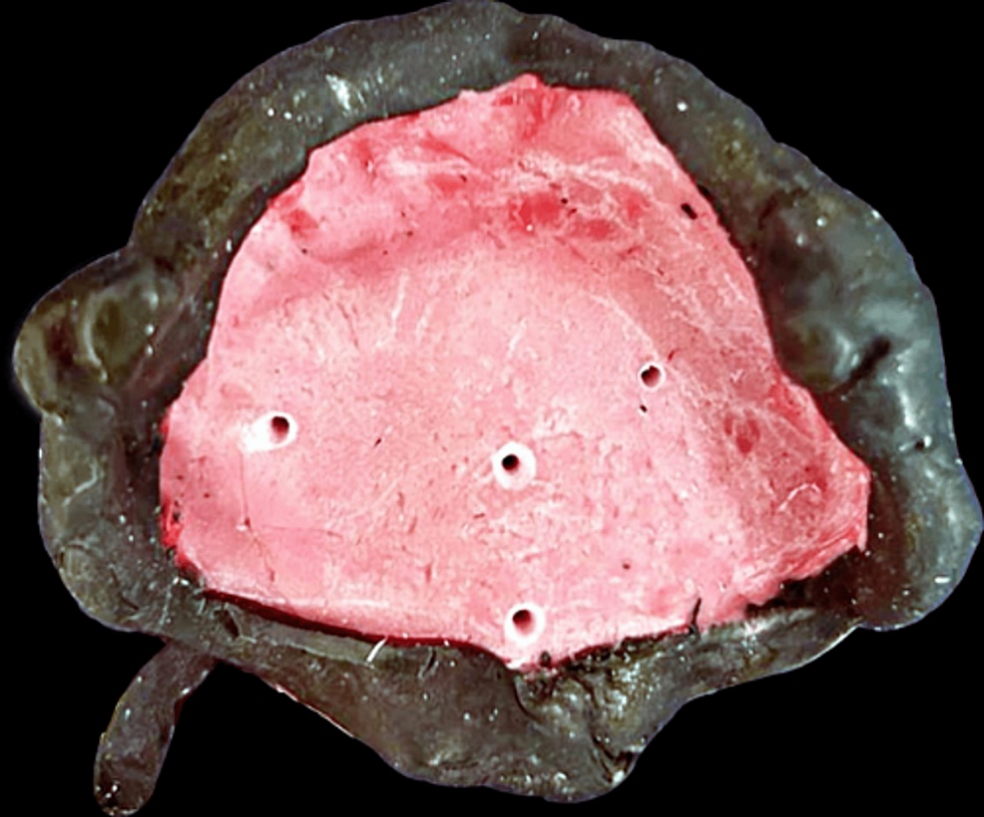
Border molding by green stick followed by recording final impression.

Blocking the undercuts allowed for the fabrication of a custom tray using autopolymerizing resin. After recording the mucosal junction by border molding with a green stick, impression material was used to create the final impression (Figure 5).

**Figure 6:**
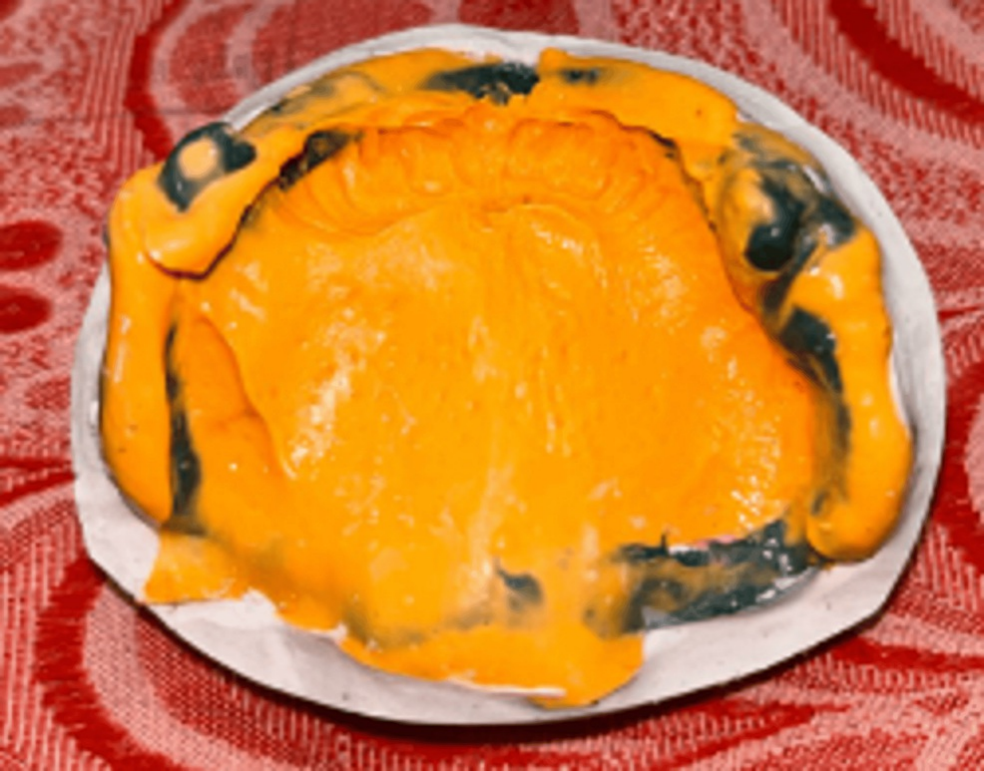
Final cast is shown in this photograph.

To obtain the final cast, all standard prosthodontic procedures including beading and boxing were performed. The impression was then poured using die stone gypsum material, and the final cast was made, as shown in Figure 6.

**Figure 7:**
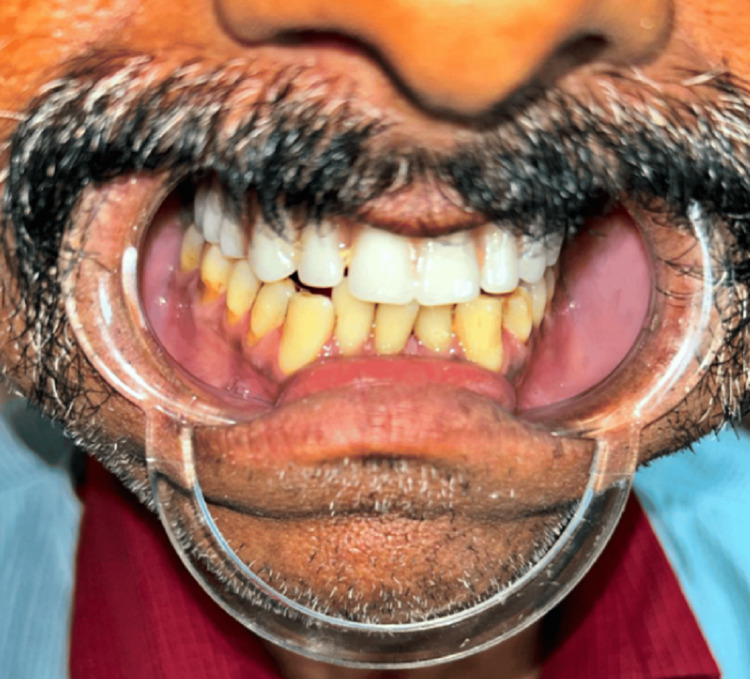
This photograph shows the try-in procedure.

Now, by using the self-cured acrylic resin, the record base was made. The occlusal rim was fabricated and bite registration was done to maintain the bilateral balanced occlusion. After biting into wax occlusal rims, the occlusion was checked. The teeth were arranged by the preexisting occlusion followed by carving and finishing of the wax by maintaining the actual anatomy. Then the obturator was tried in the patient's mouth and the try-in was done by taking the esthetic and phonetics into consideration (Figure 7).

**Figure 8:**
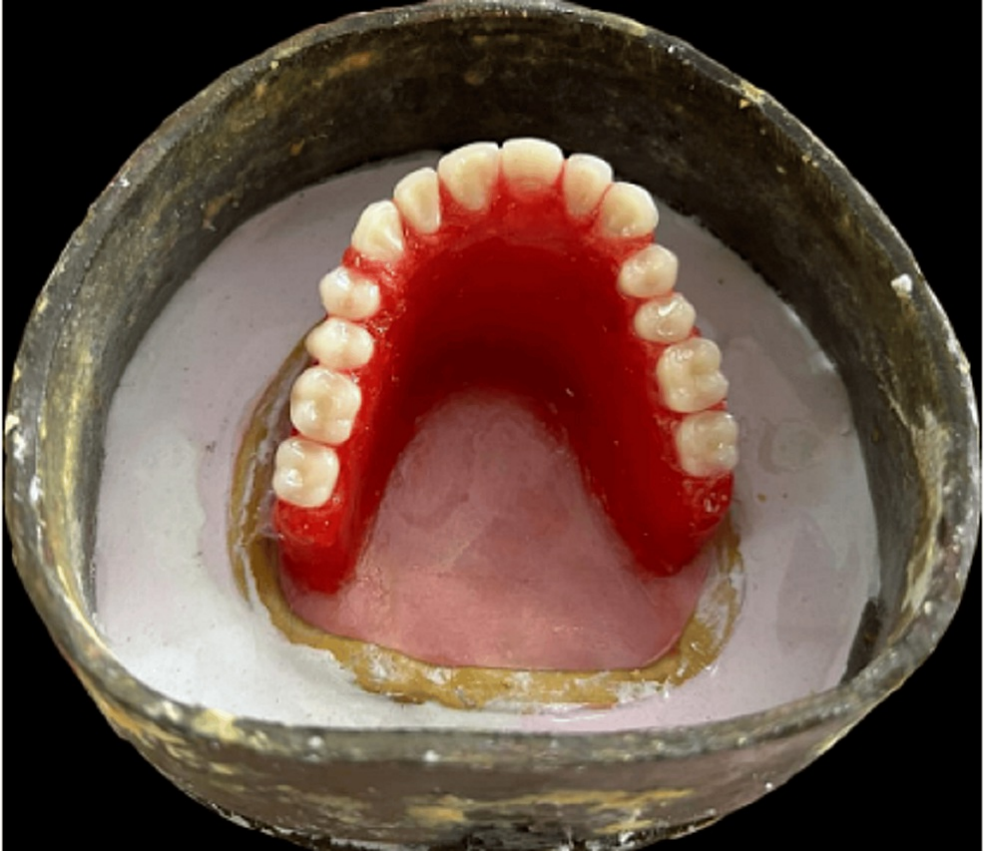
This photograph shows flasking, where the investing cast is placed with the waxed denture in a flask and dental plaster is poured so that the occlusal surface is parallel to the base.

The trial denture was sealed to the final cast using wax. Standard protocols for flasking and dewaxing were followed, and a flask investment was made, as shown in Figure 8.

**Figure 9:**
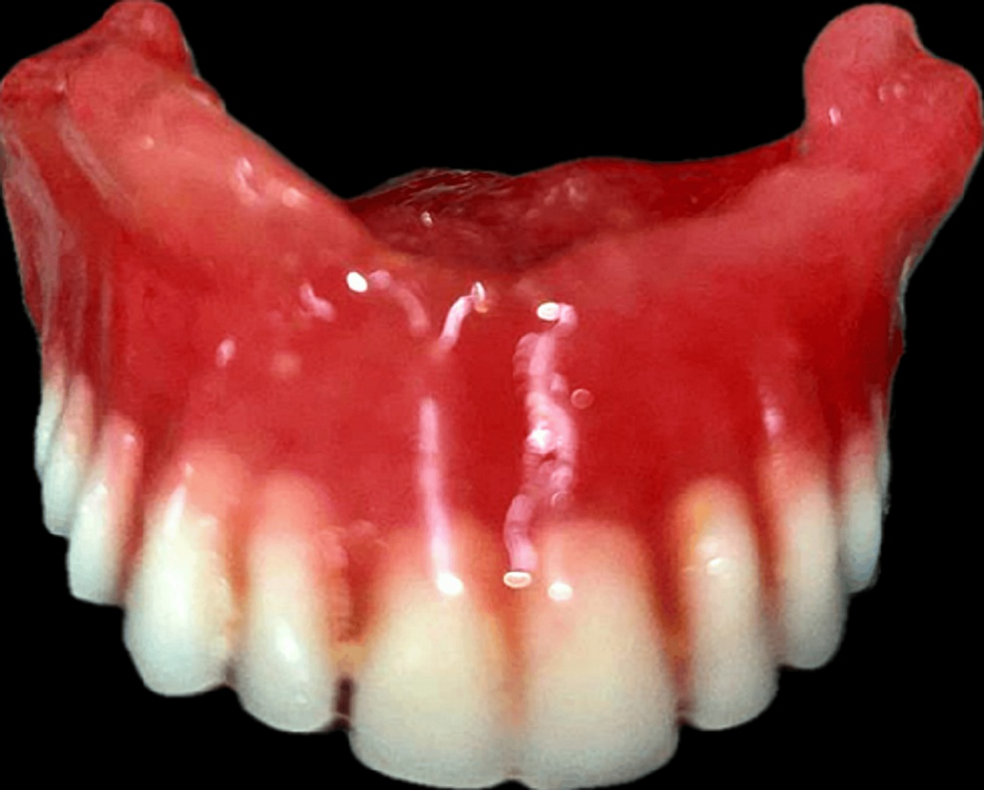
Complete fabrication of prosthesis.

During the first plaster pour, all parts other than the teeth and the waxed portion were covered with an investing stone. A 2-mm-thick coating of heat-cured acrylic resin dough was applied to the defective area. The mold was packed according to usual procedures. Processing was carried out as directed by the manufacturer. The obturator was removed during de-flasking, and complete fabrication was done, as shown in Figure 9.

**Figure 10:**
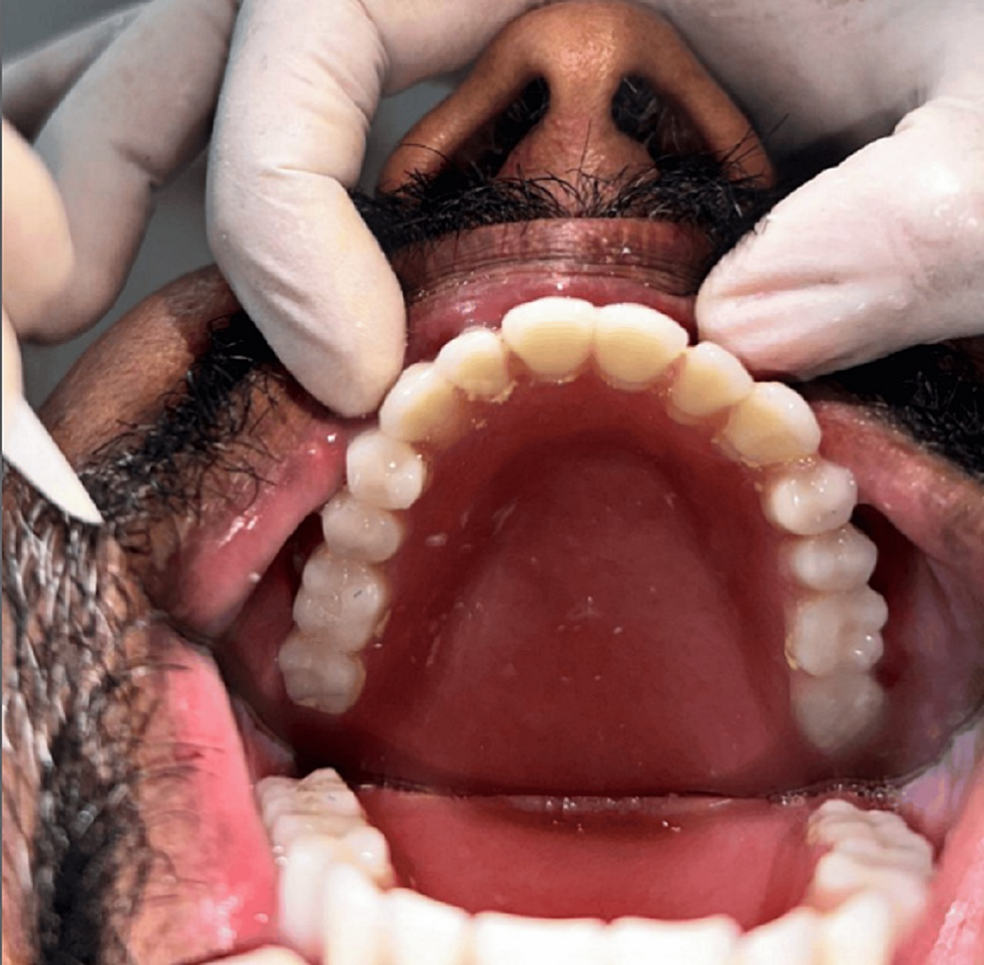
Photograph showing the delivered prosthesis.

The finishing and polishing of the prosthesis were completed. Adjustments were made based on the defect. The prosthesis was successfully delivered to the patient, meeting comfort, functional, and esthetic requirements. The patient expressed satisfaction with the delivered denture (Figure 10). The patient was recalled after one week for follow-up to recheck if there was any discomfort. To check for proper masticatory function, phonetics, and resonance of voice, the patient was recalled after three months.

## Discussion

Optimizing the functionality of the obturator prosthesis after maxillectomy is crucial for maximizing confidence and social interaction [5]. Large sections of the maxilla, or sometimes the entire maxilla, must typically be removed due to mucormycosis. To facilitate tissue recovery, modification, and stability, the conventional procedure for prosthetic rehabilitation of these patients includes immediate, interim, and ultimately definitive obturators. However, prosthodontic treatment will restore the patient's function to a normal state [6]. Definitive obturators have been recommended as a prosthodontic treatment for patients recovering from maxillectomy to preserve and regain their oral function to an acceptable degree during the postoperative phase [7]. The immediate obturator is fabricated from the preoperative impression cast, whereas the definitive obturator is fabricated from the postoperative impression cast [8]. Resection of the maxilla results in loss of speech and masticatory function, which is recovered by giving immediate obturators, whereas definitive obturators are used after the healing process is completed, which is a long-term solution. Prosthetic obturators improve life as a whole. When creating dentures for maxillectomy patients, obtaining the necessary retention to keep the denture stable in place during operation can be quite challenging. In our case, there was oroantral communication present, so there was difficulty in taking an impression on this patient. So here we used gauze to block the oroantral communication to prevent the regurgitation during the impression. In dentate patients, the obturator receives retention, stability, and support from the remaining teeth, but in this case, there was difficulty in achieving the retention due to the edentulous maxilla. As no hard tissue was present for gaining support after maxillectomy, obtaining retention, stability, and support from the remaining soft tissues is challenging. In our case, retention was gained using the soft tissue undercuts as there was no hard tissue and bone was present. Also, there was difficulty in maintaining stability, so proper bilateral balanced occlusion was established to distribute the occlusal forces evenly and prevent the dislodgment of the prosthesis. An obturator is essential to a patient's ability to regain oral function after a maxillectomy. Fabrication of a definitive prosthesis is used for maxillary defects, and proper follow-up visits should be advised to the patient [9].

However, there are many challenges in this case, particularly when producing impressions for a delayed surgical obturator. These challenges include dealing with oroantral communication, facial incisions, and factors related to healing. Additionally, full extension into the defect is not advised due to potential interference with healing, restricted oral opening, and the weight of the prosthesis, all of which can impact its retention [10]. The prosthesis should be as light as possible because the obturator's weight may function as a dislocating force [11]. Prosthesis comfort, weight reduction, and improved retention are all benefits of this appliance. An increase in the weight of the obturator compromises its functionality, making it undesirable and non-retentive for the patient. The prosthesis's weight is decreased by fabricating definitive obturators. On the other hand, definitive obturators offer reduced airway space, maximal extension, and prevention of food and fluid collection [12]. It is possible to adjust the thickness and create a definitive prosthesis that is lightweight. Educating the patient about maintaining definitive obturators and practicing good oral hygiene is crucial [13]. However, a definitive obturator is not recommended until the surgical site has healed and stabilized dimensionally, and the patient is psychologically and physically prepared for any necessary restorative therapy. For a minimum of a year, the defect's border regions will continue to undergo changes related to healing and remodeling. Rather than the bone support areas, the peripheral soft tissues are predominantly related to dimensional alterations. Fabricating definitive obturators helps improve the psychological state of patients, restores function, and provides a long-term solution that may not be achievable with other types of obturators [14].

## Conclusions

Prosthodontists need to be prepared to face the ongoing difficulties associated with prosthetic rehabilitation. Because of a highly invasive cause of mucormycosis, significant surgical resection is often required. A methodical approach to creating a permanent definitive obturator for individuals undergoing maxillectomy has been outlined. The patient's function was restored using the definitive obturator, which enhanced phonetics by producing more resonance in the voice and improved masticatory efficiency. For treatment to be effective, decisions about the extent of the maxillectomy defect and the structures to be protected are crucial. To restore speech, swallowing, mastication, and esthetics, the surgeon and the maxillofacial prosthodontist must work together as a team. Therefore, understanding the various approaches to rehabilitation for such abnormalities is vital to contribute to an enhanced quality of life.
